# Pathway-Based Drug Repositioning for Breast Cancer Molecular Subtypes

**DOI:** 10.3389/fphar.2018.00905

**Published:** 2018-08-15

**Authors:** Raúl A. Mejía-Pedroza, Jesús Espinal-Enríquez, Enrique Hernández-Lemus

**Affiliations:** ^1^Computational Genomics Division, National Institute of Genomic Medicine, Mexico City, Mexico; ^2^Centro de Ciencias de la Complejidad, Universidad Nacional Autónoma de México, Mexico City, Mexico

**Keywords:** drug repositioning, breast neoplasms, systems biology, databases, genetic, pathway analysis, personalized medicine

## Abstract

Breast cancer is a major public health problem which treatment needs new pharmacological options. In the last decades, during the postgenomic era new theoretical and technological tools that give us novel and promising ways to address these problems have emerged. In this work, we integrate several tools that exploit disease-specific experimental transcriptomic results in addition to information from biological and pharmacological data bases obtaining a contextual prioritization of pathways and drugs in breast cancer subtypes. The usefulness of these results should be evaluated in terms of drug repurposing in each breast cancer molecular subtype therapy. In favor of breast cancer patients, this methodology could be further developed to provide personalized treatment schemes. The latter are particularly needed in those breast cancer subtypes with limited therapeutic options or those who have developed resistance to the current pharmacological schemes.

## 1. Introduction

Breast cancer is a major public health concern and a main cause of death in young women worldwide (Ferlay et al., 2015). Breast cancer is characterized by an heterogeneous nature at the histological, molecular, and systemic levels. To cope with this heterogeneity, multiple prognostic and therapeutic approaches have been developed to handle this disease. From classifications based on clinical parameters or histopathologic markers (for example estrogen receptor[ER], progesterone receptor [PR], and epidermic human growth factor receptor [HER2]) (Prat et al., 2015) to molecular classifications based on gene expression values of the samples, such is the case of PAM50 classification (Parker et al., 2009).

The four breast cancer intrinsic subtypes (Luminal A, Luminal B, Basal, and Her2-enriched) defined by PAM50 offer more accurate clinical information than the classifications based on histopathologic parameters (Prat et al., 2015). The predictive power of PAM50 regarding patient prognosis is an example of this increase in accuracy (Prat et al., 2014, 2015). Furthermore, the new breast cancer international guidelines (Coates et al., 2015; Senkus et al., 2015) not only mention the utility of PAM50 intrinsic subtypes, but the latter states that they are indeed a central piece of the therapeutic and classification algorithms.

It is worth mentioning that the scarcity of effective therapeutic options, in particular for certain subtypes—such as basal, or triple-negative tumors—still present a challenge for clinicians who often have to resort to highly unspecific cytotoxic therapies.

In order to help solve this problem, theoretical and methodological advances are aimed at the development of improved therapeutic options firmly based on a deeper biological understanding of the disease. Three of these advances are particularly important in our view.

First, high throughput technologies capable of retrieve a huge amount of biological data; for example, expression microarrays or RNAseq which allow us to approach the study of the transcriptome, i.e., the set of RNA molecules present in biological entities. Second, The conception that generally speaking, a biological function of the living cell is a result of many interacting molecules; It can not be attributed to just a single gene or a single molecule. The above can be represented as a set of genes in the genome linked through a network of interacting molecules in the cell, such as a “Pathway,” representing a higher order biological function (Kanehisa and Goto, 2000). Third, the development of areas such as pharmacogenomics, which try to analyze by means of a global approach all the genes involved in the response to a certain drug in a variety of conditions (Pirmohamed, 2001). Such pharmacogenomics approach has evolved from a view centered on isolated genes to one based on pathways (García-Campos et al., 2015). Pharmacogenomics has progressively adopt a variety of high throughput technologies including genomics, transcriptomics, proteomics, and others, to enhance its capacity for generating and testing hypotheses and transfer these hypotheses to the clinical practice (Wang, 2010).

Despite these advances, for many breast cancer patients prognosis remains poor for both survival and quality of life (which is further affected by side effects of the pharmacological treatment itself). New, more effective and less harmful therapeutic options are needed, especially for certain subgroups of breast cancer patients, for example, those who suffer from basal subtype or from a tumor which develop resistance to usual therapeutic schemes (Prat et al., 2014; Yu and Jones, 2016; Friese et al., 2017). Some causes of this deficit of therapeutic options are the high economic and logistic costs involved, not only in traditional drug development (i.e., the path from concept to drug's approval), but also in the clinical evaluation of its usefulness in diseases for which the drug was not initially conceived, or drug repurposing assessment. In both cases, a more accurate selection of pharmacological targets is crucial to achieve viability and high rate of success. For all of the aforementioned reasons, a main goal of the present study is to answer the question: is it possible to identify new associations between pharmacological targets and their respective pathways to each breast cancer molecular subtype? In order to answer this, we propose a computational analysis based on the systematic inquiry of all relevant deregulated pathways specific for each breast cancer subtype, and the assessment of target genes (belonging to the aforementioned pathways), which are susceptible to pharmacological modulation. This approach relies on large, well-curated gene expression datasets from high throughput technologies coming from the two flagship projects (TCGA and METABRIC) of transcriptomic characterization of mammary tumors. Such projects are hence the gold standard reference in terms of quality and quantity of samples. Adding to this, the use of state of the art computational methods will allow us to develop a reliable, trustworthy study that supports the results that will be presented.

## 2. Materials and methods

### 2.1. Outline

**Figure 1:**
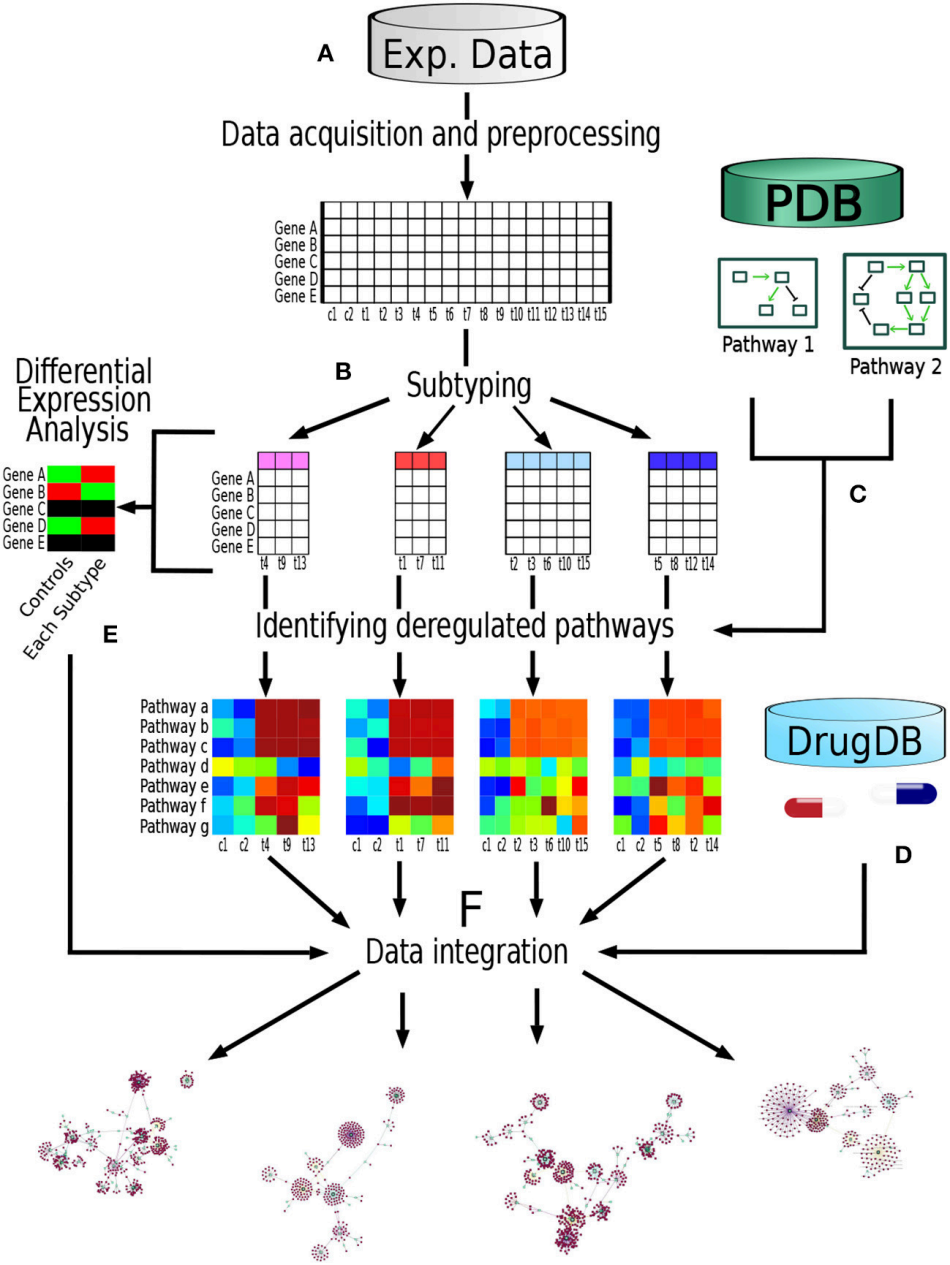
Pipeline performed in this study: **(A)** We obtained two data sets of gene expression experiments, one data set from METABRIC (Microarrays) and the other one from TCGA (RNAseq). Each dataset was analyzed independently using this pipeline. **(B)** Samples were classified in their respective molecular breast cancer subtype according the PAM50 algorithm. **(C)** From Pathway databases (PDB) we identified the most deregulated pathways in each breast cancer subtype through Pathifier algorithm. **(D)** For each pathway, genes known as pharmacological targets were identified, as well as the nature of said drug-target interaction according to pharmacological databases (DrugDB). **(E)** Differential expression analysis was performed comparing each molecular subtype against control samples. **(F)** Finally, information regarding the aforementioned steps was integrated.

The analysis pipeline followed in the present work is depicted in Figure 1. Briefly, we obtained two data sets of gene expression experiments, one data set came from METABRIC (Curtis et al., 2012) repository and the other one from TCGA (Ciriello et al., 2015). Each data set was build up trough different technologies, namely Microarrays (METABRIC) and RNAseq (TCGA). Then, the samples were classified in its respective molecular breast cancer subtype according the PAM50 algorithm (Parker et al., 2009). After that, we identified the most deregulated pathways in each breast cancer subtype through Pathifier algorithm (Drier et al., 2013). Each deregulated molecular pathway was associated to its known pharmacological targets according to information from pharmacological databases. Finally, these drug-subtype relations were classified according to information available in both the pharmacological databases as well as information from the gene expression data of the samples themselves. Supported by these analyses we develop a Database Based Drug Repurposing (DBDR) method that allows for the implementation of precision medicine approaches, that may be applied even at the individual (personalized medicine) level.

### 2.2. Data sources

As already discussed, our analysis is supported with information coming from the largest, more reliable whole-genome breast cancer gene expression datasets available, the ones provided by the METABRIC and TCGA consortia, respectively. We will briefly describe those datasets—at the level used here—. For more detailed information, please consult the original sources as referenced.

**METABRIC** (Molecular Taxonomy of Breast International Consortium) is a collection of over 2,000 clinically annotated primary fresh-frozen breast cancer specimens from tumor banks in the UK and Canada, more details about those data can be found in Curtis et al. (2012). To minimize batch effects, we only use the “discovery set” consisting in expression data from 997 samples of women with breast cancer and the “control set” a set of expression data from 144 samples from adjacent normal breast tissue.

Data were provided under request through the following download platform:

https://www.ebi.ac.uk/ega/about/your_EGA_account/download_streaming_client.

**TCGA** (The Cancer Genome Atlas) is a specialized database that stores high throughput technology data of at least 33 types of breast cancer obtained from more than eleven thousand patients. This big database is a reference point in terms of quality and sample size. We used the data set generated and published by Ciriello et al. (2015) which come from 808 biopsies of women with breast cancer and 112 from adjacent normal breast tissue.

Data were downloaded with their respective clinical information from the following internet address:

http://www.cbioportal.org/study?id=brca_tcga_pub2015#summary.

### 2.3. Preprocessing

In **METABRIC** Each RNA transcriptional profiling was extracted through the Illumina HT-12 v3 microarray platform. The consortium provides the data in the form of preprocessed expression matrices. The detailed description of the preprocessing performed in the data can be found in the original publication (Curtis et al., 2012).

Similarly to the previous case, the data used for **TCGA** are provided as preprocessed expression matrices. Details about data preprocessing could be found in the original publication (Wilkerson et al., 2014). Briefly (As reported by the author), RNA sequencing was performed on Illumina HiSeq. Resulting sequencing reads were aligned to the human hg19 genome assembly using MapSlice (Wang et al., 2010). Gene expression was quantified for the transcript models corresponding to the TCGA GAF 2.13 using RSEM4 and normalized within samples to a fixed upper quartile. Upper quartile normalized RSEM data were log2 transformed.

### 2.4. Subtyping

Samples were classified through PAM50 (Parker et al., 2009) algorithm by METABRIC and TCGA consortiums themselves. The proportions of samples per subtype inside each database are shown in Table 1.

**Table 1:** Table Distribution of subtypes by database.

**Database**	**Control**	**Basal**	**Her2**	**Lumina A**	**Luminal B**	**Sum**
METABRIC	144	118	87	466	268	1,083
TCGA	112	136	65	411	171	895
Sum	256	254	152	877	439	1,978

### 2.5. Functional enrichment analysis

To identify the most deregulated molecular pathways we use Pathifier (Drier et al., 2013). Pathifier is an algorithm that integrates experimental data from high throughput technologies and pathway information from biological databases (Supplementary Material).

Pathifier provides as a result a deregulation value for each molecular pathway in each sample. This deregulation value is called “Pathway Deregulation Score” or PDS. This methodology has been used previously to observe deregulated pathways in breast cancer samples from TCGA (Drago-García et al., 2017). Importantly, Pathifier assigns a PDS for each individual sample, which in our case is a fundamental feature for choosing this method to assess the deregulation level for pathways of each breast cancer subtype.

**Figure 2:**
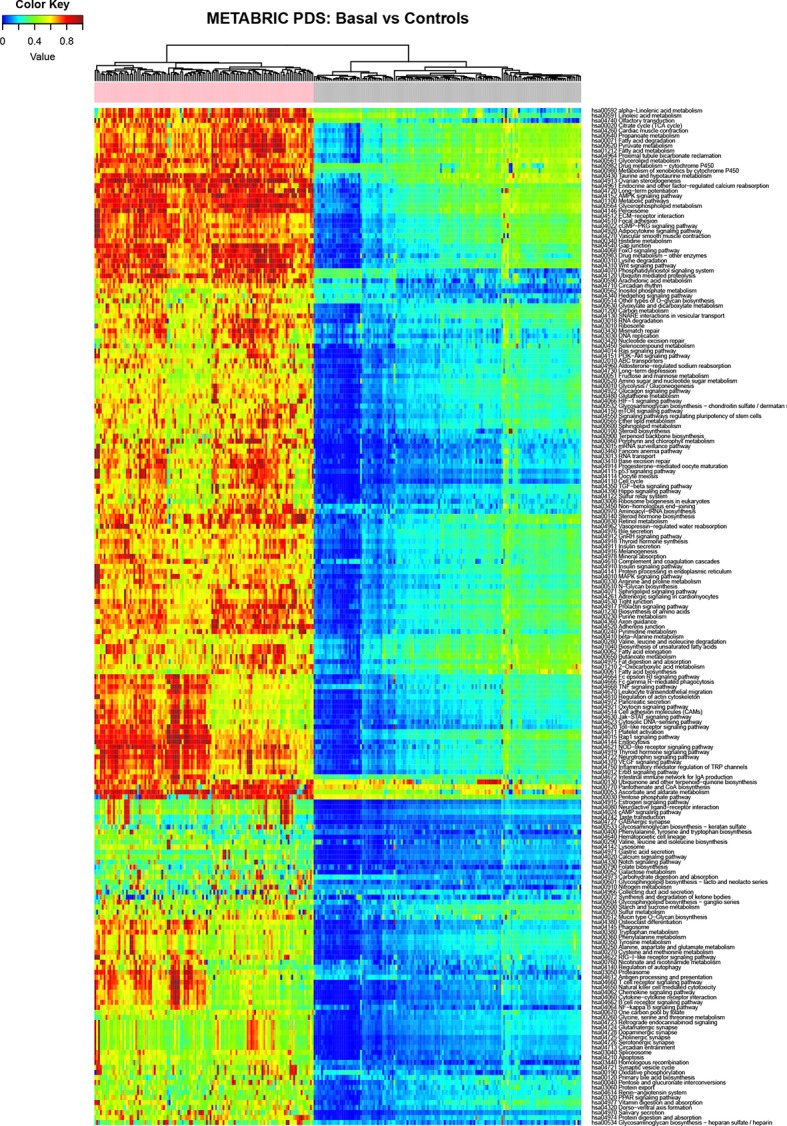
Heatmap depicting the PDS for the Basal subtype (basal samples are marked with a pink label) and normal breast tissue (gray labels) in the METABRIC study, rows correspond to molecular pathways and columns are individual samples.

In our case we use the molecular pathways defined by KEGG (Kanehisa and Goto, 2000) and the analysis was performed independently in each database (METABRIC and TCGA) for each tumor subgroup of breast cancer comparing it against its corresponding control group. Figure 2 shows the result of the calculation of Pathifier for the basal subtype of METABRIC against its respective controls.

In order to identify the most deregulated pathways by subtype we performed a z-score transformation over the PDS that belonged to each pathway(row). This transformation allow us to compare the deregulation scores (called “PDSz” here after) between different molecular pathways as described by the author of the method (Livshits et al., 2015). We looked for those pathways whose patients exhibited the highest PDSz consistently in both databases. This was done as follows: the algorithm returns “n” PDSz values (one PDSz per each patient) for a given pathway. We used the median thus obtaining only one deregulation value per molecular pathway in each database. then we sort the values and got the intersection in METABRIC and TCGA data of top 30 most deregulated pathways. Results are shown in Table 2.

**Table 2:** Concordance between the most deregulated pathways.

**Molecular subtype**	**Deregulated pathways**
Basal	Olfactory transduction, Hedgehog signaling pathway, Cell cycle, Base excision repair, ErbB signaling pathway, Neurotrophin signaling pathway, Metabolic pathways, Apoptosis, Oocyte meiosis, Drug metabolism - other enzymes
Her2	Toll-like receptor signaling pathway, Apoptosis, Vasopressin-regulated water reabsorption, Aldosterone-regulated sodium reabsorption, Glycine, serine and threonine metabolism, DNA replication, ErbB signaling pathway, Melanogenesis
LumA	Jak-STAT signaling pathway, NF-kappa B signaling pathway, Glycerolipid metabolism, Fatty acid degradation, TNF signaling pathway, Fc epsilon RI signaling pathway, Leukocyte transendothelial migration, Osteoclast differentiation, ECM-receptor interaction, Ascorbate and aldarate metabolism, FoxO signaling pathway
LumB	Steroid biosynthesis, Retinol metabolism, cAMP signaling pathway, Vasopressin-regulated water reabsorption, Adrenergic signaling in cardiomyocytes, Progesterone-mediated oocyte maturation, Thyroid hormone synthesis, GnRH signaling pathway, Glutamatergic synapse

### 2.6. Drug target associations

The aim of this procedure was to identify which of the studied *genes* (in the broad sense of the word) could be considered as pharmacological targets by virtue of a drug acting upon it, according to pharmacological databases. Drug-target associations were identified through a tool known as *The Drug-Gene Interaction Database* (www.dgidb.org). This platform integrates information of at least 15 pharmacological databases which include information about drugs, pharmacological targets, type of drug-target interaction, data sources, and other characteristics. That tool was used through its implementation as bioconductor package which name is RDGIdb (Wagner et al., 2015).

### 2.7. Prioritization of pharmacological targets

Drugs were classified according to the following criteria. First, if there was a molecular pathway with which the drug *interacts*—i.e., if the drug has any kind of physicochemical effect in at least one of the pathway's elements, having a noticeable effect in the pathway itself—. Second, if that molecular pathway resulted highly and consistently deregulated in a breast cancer molecular subtype (as it is found by applying pathway deregulation calculations in both gene expression datasets under study). Third, if the pharmacological target gene was differentially expressed in that breast cancer subtype in relation to healthy controls. Fourth, depending the type of interaction of the drug with the pharmacological target, in particular whether the effect of the drug (a) leads to a return of the target gene basal expression level (for presentation purposes,we will call homeostasis), (b) given an overexpressed/underexpressed target, leads to a higher overexpression/underexpression (anti-homeostasis), or (c) leads to an undetermined behavior of the target.

### 2.8. Differential expression analysis

Differential expression analysis is a procedure which allows to identify if the same gene holds different expression levels with respect to two conditions (in our case a breast cancer subtype compared to adjacent normal breast tissue). In this study, differential expression analysis was made through the limma (Ritchie et al., 2015) bioconductor (Gentleman et al., 2004) package for the case of METABRIC data and with DeSeq2 (Love et al., 2014) in the case of TCGA data. Threshold parameters used for those analysis were a absolute log fold chance (base 2) >1, a Benjamini-Hochberg adjusted *p*-value lesser than 0.001 and a FDR lesser than 0.1. All the code was developed using the Emacs speak statistics package (Rossini et al., 2004)

### 2.9. Individualized proof-of-concept

Since Pathifier provides a PDS per sample, the deregulation of any given pathway is different for each patient. The final section is composed by two examples of deregulated pathways in two patients with basal subtype. There we show the PDS for their most deregulated pathways, the genes which are susceptible to pharmacological modulation, and also the drugs that modulate these targets and concomitantly, the associated pathways, paying special attention to those drugs that have a mechanism of action of direct and selective interaction with the identified target, thus reducing the undesired side effects.

## 3. Results

### 3.1. Deregulated pathways by breast cancer subtype

#### 3.1.1. Deregulated pathways were consistent across different technologies

**Figure 3:**
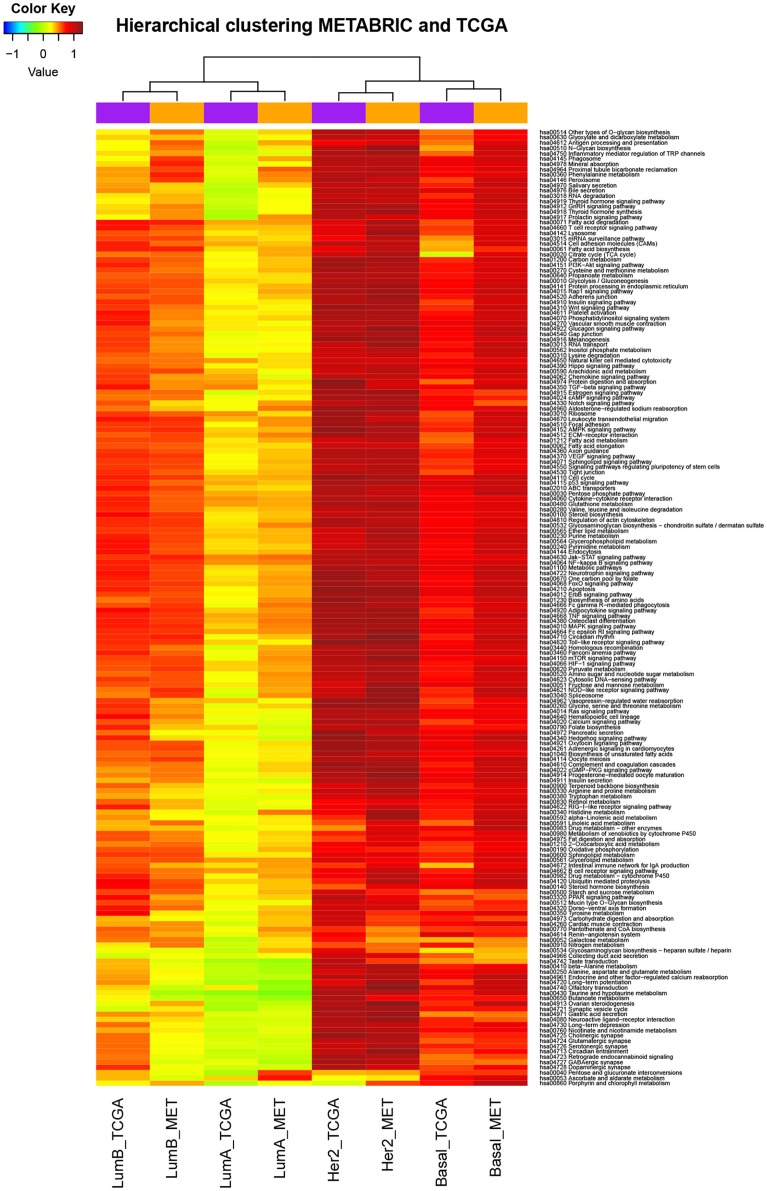
Deregulated pathways were consistent across different technologies. The data are the level of deregulation associated to each pathway in each breast cancer subtype (as is described more detailed in functional enrichment analysis subsection). Color scale corresponds to the level of deregulation in each pathway where red is very deregulated and blue is little deregulated. Rows correspond to KEGG molecular pathways, columns to subtypes of breast cancer according to the database of provenance (MET means METABRIC). We can observe that the breast cancer subtypes traditionally considered as more aggressive have higher levels of deregulation. Also we can seen that the data (sets of PDSz) do not group according to the technology by which they were obtained, instead they group according to the biological condition from which they come.

The deregulated pathways by subtype were consistent across different technologies. Unsupervised hierarchical clustering was performed to see if the set of pathway deregulation levels related to one particular breast cancer subtype was consistent with the subtype itself or instead it was consistent with the technology where the data come from. Before carrying out this procedure, were calculated the PDSz (the level of deregulation associated to each pathway in each tumoral subtype) as described in section Materials and Methods. Thus, there were eight sets of PDSz, considering four tumoral subtypes and two different data sources. After that, unsupervised hierarchical clustering was applied. The result of this analysis is shown in Figure 3, where it can be seen that data do not group according to the technology by which they were obtained, instead they group according to the biological condition from which they come.

#### 3.1.2. Agreement between the most deregulated pathways

For each tumoral subtype the 30 pathways with higher deregulation score were taken and then we selected the ones which coincided in both databases (from now on called the most deregulated pathways by breast cancer subtype). These are mentioned in Table 2.

Finally, information regarding the deregulation value of the pathway associated to the pharmacological target for the breast cancer subtype, the differential gene expression status of the pharmacological target, type of interaction between drug and pharmacological target, the relationship between the type of drug-target interaction and the differential expression status of the latter, is presented in Supplementary Material.

A brief example of the above mentioned database is provided in Table 3. Columns show several criteria by means of which the pharmacological targets were classified: *Drug* associated to the pharmacological target, *Gene* associated to the target, *Pathway* to the molecular pathway which pharmacological target belongs to. *logFC* refers to the logarithmic differential expression ratio of the pharmacological target gene in the breast cancer subtype relative to its controls. *InteractionType* to the type of interaction between the drug and the pharmacological target. *Homeostasis* is a term determined according to the differential expression status of the pharmacological target gene and the type of drug-target interaction. Finally, *Source* refers to the database from which the information was obtained.

**Table 3:** Example of the information contained in the database.

Pathway	Gene	Drug	Interaction type	Source	logFC	Effect
hsa00230 Purine metabolism	HPRT1	AZATHIOPRINE	Inhibitor	DrugBank	1.623	Homeostasis
hsa00230 Purine metabolism	PNP	CLADRINE	Inducer	DrugBank	1.628	Anti-homeostasis
hsa00230 Purine metabolism	PDE2A	TOFISPAM	Inhibitor	DrugBank	−3.962	Anti-homeostasis
hsa00230 Purine metabolism	PDE8B	KETOTIFEN	Inhibitor	DrugBank	−2.509	Anti-homeostasis
hsa00310 Lysine degradation	ALDH2	DISULFIRAM	Inhibitor	DrugBank	−2.363	Anti-homeostasis
hsa00100 Steroid biosynthesis	SQLE	NAFTIFINE	Inhibitor	DrugBank	2.703	Homeostasis
hsa00100 Steroid biosynthesis	SQLE	TERBINAFINE	Inhibitor	DrugBank	2.703	Homeostasis
hsa00100 Steroid biosynthesis	SQLE	BUTENAFINE	Inhibitor	DrugBank	2.703	Homeostasis
hsa00100 Steroid biosynthesis	SQLE	ELLAGIC ACID	Inhibitor	DrugBank	2.703	Homeostasis

### 3.2. Pharmacological targets are associated to specific molecular subtypes

Pharmacological target were selected from the pathways that are the most deregulated for a specific subtype of breast cancer and also coincide in both databases. Additionally, the target has to be differentially expressed.

The selected pathways and the number of associated pharmacological targets are shown in Table 4. There, rows are the most deregulated molecular pathways in both databases by breast cancer subtype as shown in Table 2. Columns are PAM50 breast cancer subtypes and number of pharmacological targets that are also differentially expressed in each subtype of breast cancer.

**Table 4:** Pharmacological targets by tumor subtype.

Deregulated pathway	Tumor subtype	Number of druggable targets
Adipocytokine signaling pathway	Luminal B	6
Aldosterone-regulated sodium reabsorption	Her2+	6
Apoptosis	Basal	6
Apoptosis	Her2+	3
Ascorbate and aldarate metabolism	Luminal A	1
Cell cycle	Basal	7
Cell cycle	Luminal B	4
Drug metabolism - other enzymes	Basal	3
ECM receptor interaction	Luminal A	4
ErbB signaling pathway	Basal	4
ErbB signaling pathway	Her2+	5
Fatty acid degradation	Luminal A	5
Fatty acid degradation	Luminal B	5
Fc epsilon RI signaling pathway	Luminal A	1
FoxO signaling pathway	Luminal A	6
Glycerolipid metabolism	Luminal A	3
Glycerophospholipid metabolism	Luminal B	4
Glycine, serine and threonine metabolism	Her2+	6
Jak-STAT signaling pathway	Luminal A	5
Leukocyte transendothelial migration	Luminal A	2
Leukocyte transendothelial migration	Luminal B	4
Melanogenesis	Her2+	2
Metabolic pathways	Basal	46
Neurotrophin signaling pathway	Basal	7
NF-κ signaling pathway	Luminal A	1
Olfactory transduction	Basal	1
Oocyte meiosis	Basal	5
Osteoclast differentiation	Luminal A	3
Osteoclast differentiation	Luminal B	7
Steroid Biosynthesis	Luminal B	1
TNF signaling pathway	Luminal A	5
Toll-like receptor signaling pathway	Her2+	5

*Notice that despite there are subtypes that present the same deregulated pathway, the number of targets between subtypes is different*.

**Figure 4:**
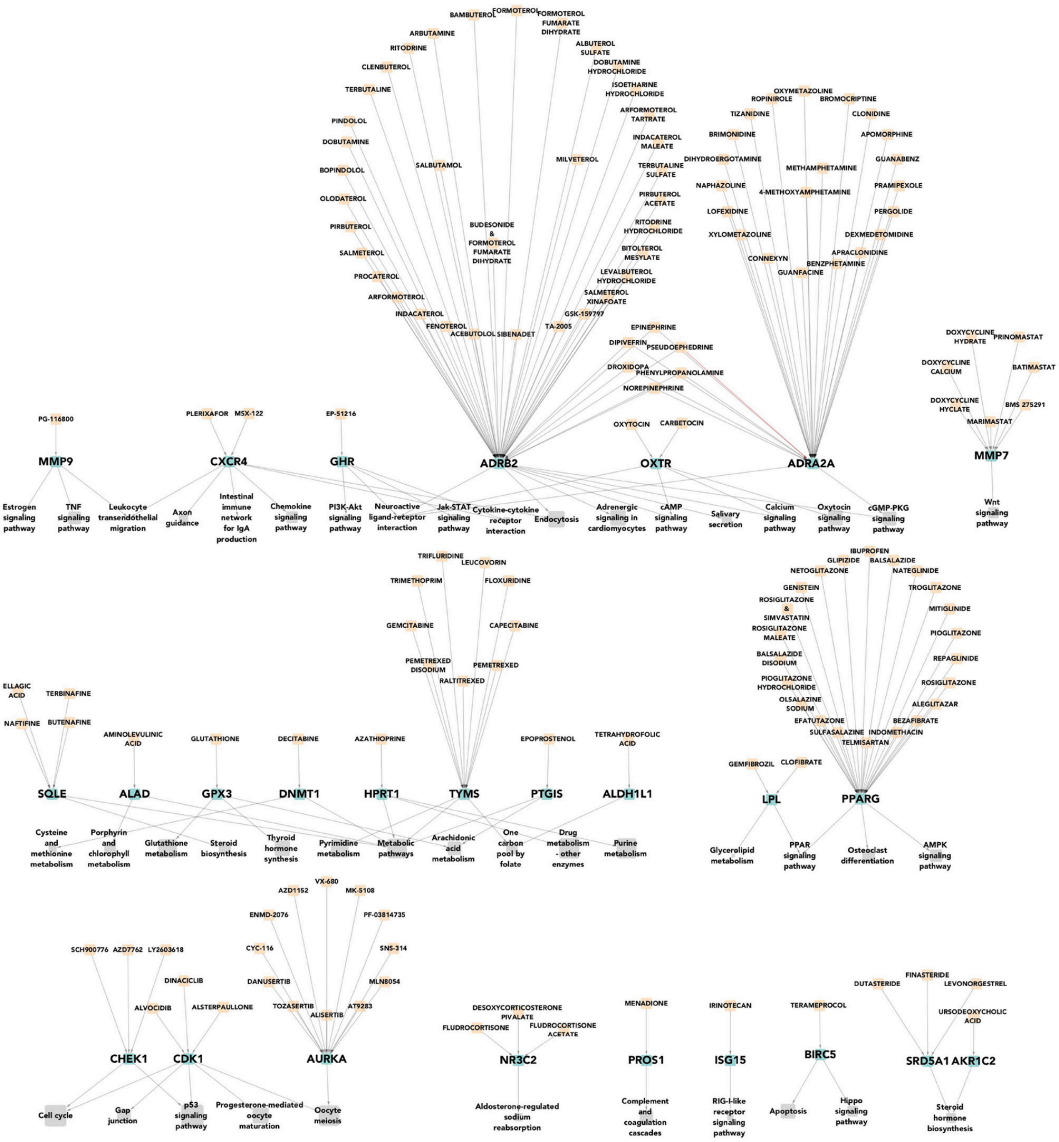
Pharmacological targets of the most deregulated pathways for basal breast cancer subtype. In gray the most deregulated molecular pathways by PDS are shown. In green color, we represent the differentially expressed pharmacological targets that had a drug which lead to homeostasis. Finally, in apricot color we show the drugs that affect expression of such pharmacological targets. We can observe that there are many more pharmacological targets associated to this specific tumoral subtype than those currently used for their clinical treatment.

We can notice that there are many more pharmacological targets associated to each molecular subtype than those currently used for their clinical treatment. In Figure 4 we can see a multipartite network of pathways-targets-drugs for basal tumors. A number of potential therapies (not currently used) is evident, as is the redundancy of the network, which could be indicative for flexible therapeutic approaches.

Seventy-nine drugs leading to homeostasis over eight highly deregulated pathways have been found for the Basal breast cancer subtype. This acquires particular relevance since this subtype is closely associated to triple negative tumors, for which directed (i.e., non-purely cytotoxic) pharmacological therapies are scarce.

The fact that a high number of drugs already in the market (i.e., after passing phase I, II, and III clinical trials and receiving legal approval) are able to target directly some of the most deregulated pathways in this hard-to-treat tumors points out to the actual scope of pathway-based drug repurposing approaches. A further application of these findings on the road to personalized medicine is shown in the Discussion section.

## 4. Discussion

Breast cancer is a public health problem which treatment require new pharmacological options. In the present study, For each breast cancer molecular subtype we looked for deregulated pathways that are pharmacologically modulable and are not currently used in the corresponding breast cancer subtype therapy. We have identified new pharmacological targets (and their respective pathways) that are potentially useful in breast cancer subtypes treatment.

Significant amount of work has been dedicated to the discovery of novel drug-disease associations. Previous efforts have been made to associate drug response with gene signatures (Lamb et al., 2006; Lee, 2017). However, those tools were designed to be used in cell lines, their performance in real human cells has not been explored. Furthermore, those approaches were built up with a relatively small number of drugs, and they were not built specifically in the context of breast cancer.

With this in mind, we performed a data-driven approach to the actual phenomenology of the different breast cancer subtypes as represented by their distinctive gene expression patterns, to look up for sets of patient-specific deregulated pathways, then by resorting to molecular and drug databases. We designed a methodology to find out druggable targets whose associated drugs were already in the market, i.e., we built a drug repositioning strategy.

### 4.1. New relations between pathways and subtypes

Table 2 shows the set of the highly deregulated molecular pathways associated to each subtype of breast cancer. The description of how the associations were made is discussed in the section Materials and Methods, as well as in the Results section. Some of the associations found in the present study between breast cancer subtypes and molecular pathways are already known and intensively studied, in fact, there are pharmacological targets belonging to these molecular pathways which are currently employed for the treatment of the corresponding breast cancer subtype. Some of these relationships will be mentioned below.

With regard to Her2-enriched subtype, in our study, it is associated to the ErbB signaling pathway one of the most studied pathways for this subtype (the name of this subtype of breast cancer is due to the relationship with this pathway). The ErbB2 receptor is the mainstay of pharmacological treatment in patients with the Her2-enriched breast cancer subtype (Senkus et al., 2015). In the present study we also associate the Her2-enriched subtype with the molecular pathway of apoptosis and DNA replication, where it has already been described in the literature to detail how the overexpression of Her2 leads to uncontrolled cell proliferation and suppression of apoptosis (Carpenter and Lo, 2013).

Regarding the basal subtype, its association with the molecular pathways of apoptosis and cell cycle is not new; the increase in cell proliferation and apoptosis is a well-known characteristic of this subtype (Choo and Nielsen, 2010). Moreover, lapatinib (a Her2 inhibitor) has been shown to be useful in treatment when combined with other agents in breast cancer models of the basal phenotype (Liu et al., 2011), therefore the association of this molecular subtype with Her2 signaling pathway is not entirely new.

Although several associations found in the present study between molecular pathways and subtypes of breast cancer are under intense study, here we propose novel associations that do not have been previously reported, based on the present study. This strategy results promising to evaluate their usefulness in generating knowledge regarding breast cancer treatment.

### 4.2. New relations between pharmacological targets and subtypes

**Figure 5:**
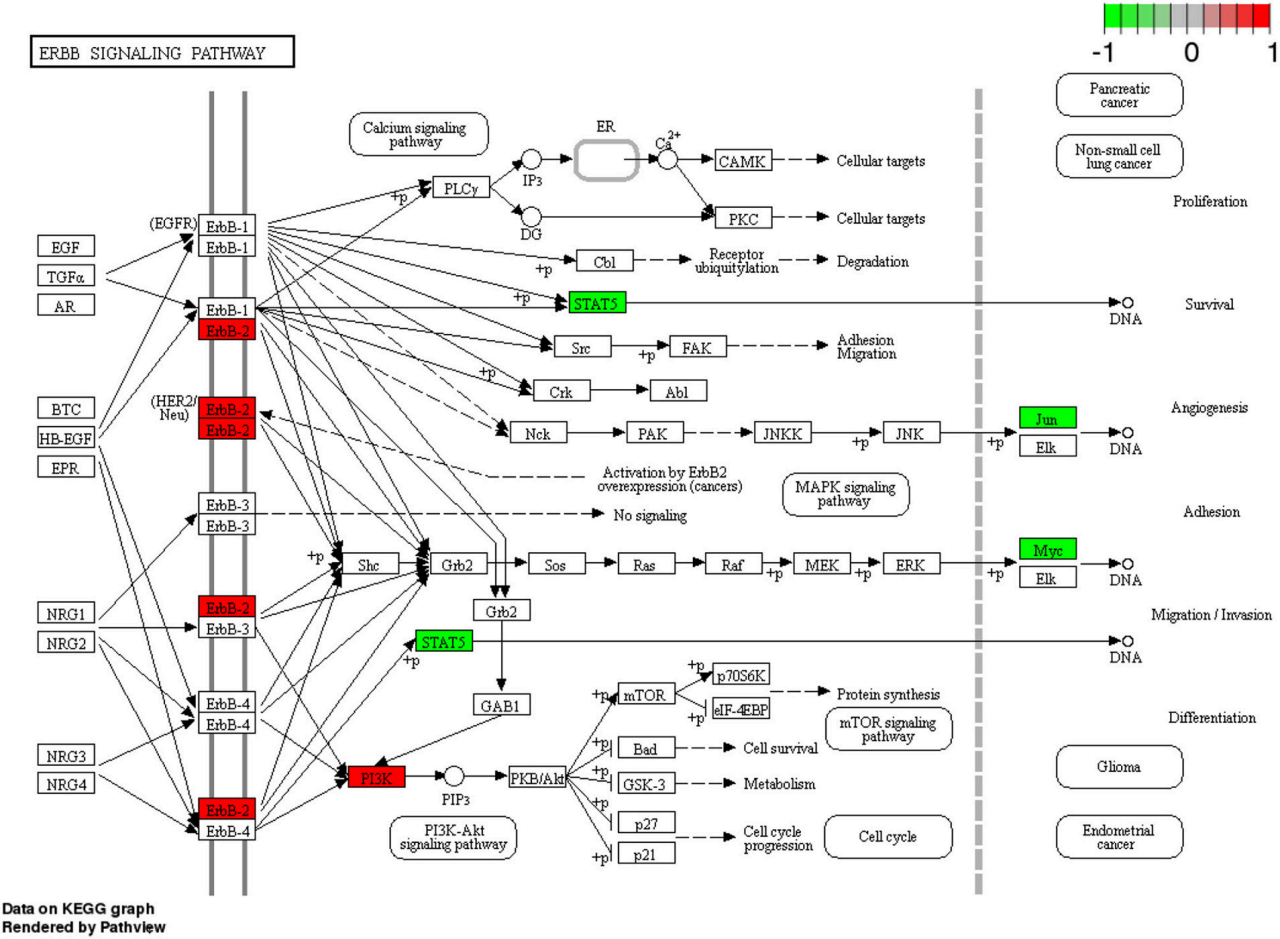
Pharmacological targets of ErbB2 signaling pathway in Her2-enriched subtype. The figure shows the five differentially expressed pharmacological targets (more than those currently exploited in the clinic) obtained with our approach.

We found different pharmacological targets associated to each breast cancer subtype. The number of pharmacological targets associated to each breast cancer subtype is shown in Table 4 and how they were associated is described in the Results section. In that table we can note that even in the cases where two breast cancer subtypes share the same deregulated molecular pathway, the number of differentially expressed pharmacological target genes is different. By observing Table 4 we see that in the molecular subtype Her2-enriched the molecular pathway “ErbB signaling” has five deregulated pharmacological targets. These are shown in detail in Figure 5. However only one of these five pharmacological targets is currently indicated in the clinical practice guidelines for breast cancer: ErbB2.

Currently, 1–5 pharmacological targets are typically used in clinic, depending on the molecular subtype of the patient. In this work we show that more than a dozen pharmacological targets are associated to each breast cancer subtype. Therefore, a subsequent evaluation of these new pharmacological targets may lead to some new therapeutic options, needed in the clinic environment. Finally, it is worth to mention that each pharmacological target may have several (up to dozens) drugs or chemical compounds that exert actions over it. Such condition amplifies the ways to modulate the therapeutic effect.

### 4.3. DBDR in the age of personalized medicine

In what follows we will present, as a *proof-of-concept*, an application of the DBDR approach to two individual breast cancer patients (within our datasets) with known gene expression profiles. Based on such expression profile, specific molecular subtype and main deregulated pathways can be known.

We decided to focus these examples in the basal subtype, since the scarcity of non-cytotoxic therapeutic options and the poor prognosis of patients having this phenotype. In the two following boxes we show the deregulated pathway with their respective PDS, their associated target genes, and the drugs that lead to homeostasis of said pathways. Each proposed drug set was checked so that all of the drug pairs do not have any known drug interaction. Finally we discussed a personalized set of drugs for each individual.

#### 4.3.1. Individual therapy designed for patient [MB.3058]

This is a patient classified as having a basal tumor with the following deregulated pathways:

**Table:** 

Pathway	PDS	Target	Drug
Steroid biosynthesis	0.937	SQLE	TERBINAFINE
Pyrimidine metabolism	0.466	TYMS	PEMETREXED or Capecitabine
Sphingolipid metabolism	0.628	SPHK1	SK1-I
Olfactory transduction	0.736	NA	NA
Apoptosis	0.398	BIRC5	ALVOCIDIB or Paclitaxel
Oocyte meiosis	0.406	AURKA, CDK1	ALISERTIB, DINACICLIB
Cell cycle	0.328	CDK1	DINACICLIB
Neurotrophin signaling pathway	0.765	NA	NA
ErbB signaling pathway	0.783	NA	NA
Drug metabolism–other enzymes	0.712	NA	NA

In view of this particular set of deregulated pathways and gene expression patterns, *individualized therapy* for this patient may include the following drugs: **TERBINAFINE**, **PEMETREXED**, or **CAPECITABINE**, and **SKI-1**. Alvocidib, paclitaxel, alisertib y dinaciclib are not considered since the low PDS in their respective pathways.

#### 4.3.2. Individual therapy designed for patient [MB.5387]

This is a patient classified as having a basal tumor with the following deregulated pathways:

**Table:** 

Pathway	PDS	Target	Drug
Steroid biosynthesis	0.597	SQLE	TERBINAFINE
Pyrimidine metabolism	0.605	TYMS	PEMETREXED or Capecitabine
Sphingolipid metabolism	0.590	SPHK1	SK1-I
Olfactory transduction	0.500	NA	NA
Apoptosis	0.712	BIRC5	ALVOCIDIB or Paclitaxel
Oocyte meiosis	1	AURKA, CDK1	ALISERTIB, DINACICLIB
Cell cycle	0.718	CDK1	DINACICLIB
Neurotrophin signaling pathway	0.839	NA	NA
ErbB signaling pathway	0.616	NA	NA
Drug metabolism–other enzymes	0.846	NA	NA

In view of this particular set of deregulated pathways and gene expression patterns, *individualized therapy* for this patient may include the following drugs: **TERBINAFINE**, **PEMETREXED**, or **CAPECITABINE**, **SK1-I**, **ALVOCIDIB**, or **PACLITAXEL**, **ALISERTIB**, and **DINACICLIB**.

### 4.4. Scope and limitations

A systems biology approach such as the one presented here is aimed at primary discovery and hypotheses generation. As such, there is a need to test the findings obtained here—in the context of the pharmacogenomics and lifestyles of actual patients—before they can be fully beneficial in a general clinical practice.

Considering the previous comments, we cannot stress enough that this theoretical approach is still far from constituting an actual aid to the clinician. For this reason, no further considerations on dose, drug combinations and patient features (other than tumor subtype, gene expression profile and deregulated pathways) are being considered.

## 5. Conclusions

New molecular pathways and their respective pharmacological targets of potential therapeutic utility were associated to each of the different breast cancer molecular subtypes. This allows subsequent focused assessment of the pharmacological targets associated to each subtype in order to find new therapeutic options for the treatment of patients suffering from this disease. The knowledge derived from this approach will result particularly useful for those patients that have developed pharmacological resistance to canonical treatment and/or suffer from tumors with reduced therapeutical options, such as basal or triple-negative breast cancers.

Here we present an application of pathway-based analyses based on the detailed study of two of the largest and more trustworthy international cohorts, including whole genome high quality transcriptomic data, coming from either RNASeq or microarray technologies: The METABRIC and TCGA collaborations. By analyzing high throughput gene expression data for thousands of patients, using a robust probabilistic approach to find the most deregulated pathways for each subtype (even at the per-patient level) and assessing the findings by crossing-out the information with highly curated knowledge-based drug target databases, we have been able to develop a methodology that allows for a detailed drug-repurposing scheme to treat the different breast cancer tumor types and that, in principle (as an introductory proof-of concept) may even be extended up to the single patient design, thus paving the way to personalized medicine to treat such complex pathologies as breast cancer using a pragmatic drug re-use approach.

Hopefully, the work presented here will contribute to save considerable amounts of financial resources—by resorting to drug repurposing strategies—, but above all, may soon allow us to save more and more lives of people suffering from this excruciating disease.

## Author contributions

RM-P carried out analysis and calculations, devised code, and database management duties and contributed to the writing of the manuscript. JE-E contributed to the discussion, reviewed analysis and contributed to the writing of the manuscript. EH-L designed and coordinated the project, contributed to the discussion, supervised, and reviewed the writing of the manuscript. All authors read and approved the final manuscript.

### Conflict of interest statement

The authors declare that the research was conducted in the absence of any commercial or financial relationships that could be construed as a potential conflict of interest.
